# Advances in Residential Design Related to the Influence of Geomagnetism

**DOI:** 10.3390/ijerph15020387

**Published:** 2018-02-23

**Authors:** Francisco Glaria, Israel Arnedo, Ana Sánchez-Ostiz

**Affiliations:** 1 Theory and Design Department, School of Architecture, University of Navarre, 31009 Pamplona, Spain; 2 Electrical and Electronic Engineering Departments, Public University of Navarre, 31006 Pamplona, Spain; israel.arnedo@unavarra.es; 3 Building Constructions, Services, Structures Department, School of Architecture, University of Navarre, 31009 Pamplona, Spain; aostiz@unav.es

**Keywords:** healthy building, prevention residential uses, building design

## Abstract

Since the origin of the Modern Movement, there has been a basic commitment to improving housing conditions and the well-being of occupants, especially given the prediction that 2/3 of humanity will reside in cities by 2050. Moreover, a compact model of the city with tall buildings and urban densification at this scale will be generated. Continuous constructive and technological advances have developed solid foundations on safety, energy efficiency, habitability, and sustainability in housing design. However, studies on improving the quality of life in these areas continue to be a challenge for architects and engineers. This paper seeks to contribute health-related information to the study of residential design, specifically the influence of the geomagnetic field on its occupants. After compiling information on the effects of geomagnetic fields from different medical studies over 23 years, a case study of a 16-story high-rise building is presented, with the goal of proposing architectural design recommendations for long-term occupation in the same place. The purpose of the present work is three-fold: first, to characterize the geomagnetic field variability of buildings; second, to identify the causes and possible related mechanisms; and third, to define architectural criteria on the arrangement of uses and constructive elements for housing.

## 1. Introduction

The purpose of this research is to establish architectural criteria in the design of residential buildings, with prevention in terms of minimizing human exposure to variations in the Earth’s magnetic field as the principle focus.

The advances and requirements in terms of energy efficiency, acoustic insulation, materials, life cycle, and facilities are such that building quality has risen to a level that any housing developer already considers irrevocable. However, regarding the issues addressed in this paper, progress has been minimal. There are no standards, even at the recommendation level, in the field of architecture that allow us to rethink the arrangement of uses in relation to exposure to the geomagnetic field. From the health and safety side, during a time when issues such as geopathies [1] or sick building syndrome [2] are being recognized, we understand that it is time to investigate these issues in a cross-sectional way. People’s well-being and health are objectives to be pursued in all architectural actions and must be prioritized over the wants of corporations and particular interests.

This paper assesses the influence of the terrestrial geomagnetic field in a residential building, with the purpose of prioritizing the habitability and health of the people living in residential buildings in architectural design. Architects are largely responsible for defining how and where people will use spaces, especially resting places; thus, the implications of their decisions must be understood. We aim to analyse the location of bedrooms in the building design, due to the probable influence of geomagnetic field variability on the health of its occupants. Given that other disciplines are establishing connections between natural radiation and its possible effects on human health, a necessary channel could be opened to analyse this issue and to establish a cross-sectional relationship between the two fields of knowledge.

The magnitude of study is related to the detection of potential health risks, during the time we rest inside residential buildings. Our essential data is based upon over published diagnosis for the competent organisms [3]. Other studies point to the implications of the rise of the expectancy of life [4], or adverse health effects for 15% of the population. Due to notable geomagnetic variations [5] or concerning the causes of such illnesses like cancer [6], where we find a 5% total can be produced by physical factors, due to radiation from the sun and the earth.

New studies are necessary when addressing long-term exposures [7]. Given the growing and unstoppable environmental debate in residential construction to promote more sustainable buildings [8,9] preventative healthcare must be at the centre of the debate, as it is a socio-economic concern in many countries [10].

First, norms and recommendations are analysed to define the bases of a reference dosimetry [11,12,13]. Afterwards, the technical standard SBM, Standard of Building Biology 2015, by the Baubiologie Institute of Germany IBN [14] is adopted and interpreted as a guide for the risk prevention of exposure to geomagnetic field variability expressed in nanoteslas (nT), distinguishing four degrees of possible effects on human beings:

- Non-significant values    0–100
- Weakly significant     100–200
- Strongly significant     200–1000
- Extremely significant    +1000 nT

For the analysed dosimetry, we set values close to 1 µT (1000 nT), since it requires immediate and rigorous action based on the precautionary principle. The values are intended for resting areas associated with long-term risks [15,16] so it is a question not of determining the value of the Earth’s magnetic field in the building but of specifying the values obtained for comparative purposes and analysing the causes of the variation.

Second, we establish the principle that our work does not intervene in the study of electromagnetic fields produced by artificial origin sources [17].

Third, we are aware that the choice of the case study is decisive. That is why a residential tower with 81 dwellings was chosen, built in 1980 in Pamplona (city in northern Spain), since it is a building with a typology and a constructive-structural system that we can qualify as standard. In this way, the results can be analysed, compared, inferred, and extrapolated through a sufficient statistical sample size. The building is on land that has groundwater, given the existence of drainages in the foundation, and it has a pumping well exclusively designed for it, located on the lowest floor of the building.

The World Health Organization (WHO) [18] recommends more research in the case of static magnetic fields, because the level of information on long-term delayed effects is currently insufficient. With large gaps in the knowledge about the influence on people [19], most national standards are based on the recommendations of the International Commission on Non-Ionizing Radiation Protection [20]. This non-governmental organization, recognized by the WHO [21] evaluates the results of scientific studies carried out all over the world, and based on an in-depth analysis of all the publications; it elaborates guidelines that establish recommended exposure limits. These criteria are also adopted by Resolution 1815/2011 CEE, applying the principle As Low As Reasonably Achievable (ALARA) [22]. The Directive 2013/35/UE of the European Parliament, which establishes values according to frequencies, also applies this principal, although prolonged exposure over time is not taken into account. It also proposes more investigations. 

## 2. Status of the Situation

Since one third of our life is spent sleeping, sleep is one of the vital functions of our body. Nocturnal resting, which is associated with circadian cycles, is our main repairing method [23] and is essential for carrying out our daily activity. It allows us to maintain the connections between neurons for the consolidation of memory. It is the necessary recovery our bodies need for the reconstitution of energy reserves. Sleeping well is associated with good health. This study analyses the geomagnetic disturbances that can alter the conditions of a good night’s sleep [24]. Based on studies collected by the World Health Organization [25], the knowledge on the relationships between low frequency electromagnetic fields should be expanded.

Considering that exposures to electric and magnetic fields, outside of extremely low frequencies, act independently from each other, they should be treated separately from the induction of electric fields and currents within the human body. Although human beings have the capacity to adapt to the environment in which they live, a prolonged stay in the same place can generate pathologies in people from long-term exposure to remarkable variations of the geomagnetic field. The incidence depends on personal sensitivity, the type and dose of natural radiation, the time of exposure or synergy with other factors such as sedentary life or type of food, and prevention is recommended as a general principle [26].

Several authors state that the geomagnetic alterations in buildings should be analysed according to the location in the dwelling, as well as the daily time of personal exposure to the magnetic field in the interior of buildings [27]. This may be related to the discovery of magnetite crystals in human tissues [28,29] given that their concentration is high enough to respond to the variations of weak magnetic fields [30]. To analyse the direct interaction of these fields within the human body during nocturnal rest, we turned to experts in the field, who introduced us to an extensive debate. Some are of the position that these fields impact health conditions based on the effects of electric fields induced on radical pairs [31], on magnetite cell regeneration, and Schumann waves. Other authors argue that human beings detect the geomagnetic field, although its variations are not biologically significant [32]. Since our position is preventive, we rely on experts with the same attitude and do not participate in the debate on the degree of health conditions [33,34]. We chose to determine the influence from dosimetry and propose design alternatives. 

First, the static magnetic field can affect radical pairs [35,36]. The direct effect of a magnetic field on the recombination rate of radical pairs [37] is an example of the mechanism that could play a role in sensitivity to the magnetic field, given the importance of radical pairs in biological systems. The probability of radical pair recombination according to their rotational state is significant and is affected by the direct-current magnetic field. 

Second, cells are capable of synthesizing magnetite particles that can align themselves with the geomagnetic field [38] due to the way that human cells regulate themselves and the influence of magnetite on magneto-sensitive proteins [39], as well as the effects of bio-mineralization and magnetoreception in the body [19]. Therefore, living beings and especially human beings require geomagnetic activity [40].

Third, the relationships between the geomagnetic field and cell regeneration [41], which are related to the nocturnal reduction of melatonin [42] and the activity of the pineal gland [43], have been investigated [44].

Fourth, there is a relationship between the biological mechanisms of the human being with Schumann waves and a geomagnetic field [45]. This phenomenon was named in honour of Winfried Otto Schumann [46], who mathematically predicted its existence in 1952, despite being detected for the first time by Nikola Tesla [47]. The first spectral representation of this phenomenon was conducted by Balser and Wagner in 1960 [48]. The relationship is due to the resonant interaction with the neurons and the possible alteration of the melatonin-serotonin ratio [49]. Since the lowest frequencies of Schumann waves and the most intense ones are close to 7.8 Hz, it is similar to the dominant frequency of the human brain, 10.5 Hz [5] and similar to the frequency of the Earth’s core.

### 2.1. The Geomagnetic Field

Therefore, from the geomagnetic field point of view, this is the sum of an internal field due to the action of the Earth as a permanent magnet and an external field generated in the environment by factors such as atmospheric and solar activity. The Earth’s internal magnetic field has its origin in the electric current, which circulates through the upper layer of the Earth’s core [50]. The daily fluctuation of the Earth’s magnetic field is from 10 to 100 nT [51]. The main field varies slowly over time and can be described by mathematical models such as the International Geomagnetic Reference Field and World Magnetic Model (WMM) [52].

The variations in natural magnetic fields can be triggered by internal causes or by external disturbances. It is estimated that 90% of the magnetic field value originates from the core of the Earth, with values of 19,000 to 55,000 nT, depending on the geographical zone. It is necessary to emphasize the difference in magnitude of magnetic field values. In the magnetosphere and ionosphere, values oscillate between −79 and 26 nT [53]. Therefore, we study the geomagnetic values originating from the terrestrial nucleus due to the magnitude of its magnetic field and the influence on the human body. 

The intensity of the magnetic flux increases or decreases within local limits, and terrestrial radiation is also altered by other artificial factors related to the design and construction of buildings [54]. In turn, the general buffering effect of a resistant structure that occurs in all buildings [55] must be taken into account to adequately establish the comparison values of this study. 

In addition, we must consider the influence of magnetism on a prone human body, which is the usual position of a body in bed. As noted in the monograph of the International Commission on Non-Ionizing Radiation Protection IARC [56] in volume 80 part 1 regarding non-ionizing radiation, a geomagnetic field perpendicular to the torso provides the greatest induced quantities.

### 2.2. The Soil

Regarding the type of soil, all terrain types are susceptible to transmitting the geomagnetic field, although it also influences water content and any magnetic minerals present [57]. At the same time, water occupies 3/4 of the Earth’s surface, of which 97.1% is salt water and 2.9% is fresh water. From this volume of fresh water, 75% is glaciers, 24.7% is underground streams, and 0.3% is lakes, reservoirs and rivers. If we add the contribution of rainwater to underground channels, the probability of finding water in the subsoil at any depth is considerable [58].

It should be taken into account that the fact for alteration of the geomagnetic field, the depth of the water current does not matter, as well as the fact that the type of terrain in combination with underground streams influences the geomagnetic field differently, with calcareous soils having a weaker influence than siliceous, quartzite, or granite [59]. Circulating underground streams produce an electrical current, which depends on the speed and type of terrain through which it circulates, releasing an electric potential that generates a vertical electromagnetic field that modifies the natural field of terrestrial energy [60]. 

In addition, the existence of fissures, cracks, or faults, which are manifested on the surface as ruptures of terrestrial electric and magnetic fields, can be dry or wet if they channel circulating water, where the possible effects increase. Faults are breaks in the Earth’s crust that create raised or sunken systems. They can be normal or inverse, have horizontal displacement, be Horst and Graben type or be compression faults. 

This study does not aim to locate water streams or to define the types of faults or the causes of the modification of terrestrial energy. We are dedicated to measuring the existing levels of the geomagnetic field and the percentage of variation based on the monthly indices of pluvial flow, which have been calculated proportionally with respect to the annual total referenced in Pamplona [61].

### 2.3. Architectural Paths

Regarding residential construction, we have the responsibility of creating habitable spaces, and therefore, the health and comfort of people need to incorporated as a parameter in the design first. The building should have all its stylistic and functional attributes designed based on the scheduled needs and mandatory regulations.

To establish architectural rules, in the process of adapting as history develops, we must incorporate simple and effective solutions related to the placement of bedrooms in dwellings. The implementation of these criteria does not hinder or imply any imposition of style on a building. In fact, it is negligible conceptually and visually, although its influence is appreciable for a long-term occupant.

During the last twenty years, a sensitivity towards sustainable architecture has been progressively incorporated into our profession, very focused on reducing the impacts that buildings produce on the environment. In addition, the criterion that buildings and their surroundings have to provide healthy and comfortable spaces for users, as promoted by the Living Building Challenge and Well Building standards [62], is becoming stronger. The placement of spaces in buildings where occupants may linger for longer periods is largely tasked to architects, although they are not the only ones. The reference book in architecture and engineering [63], in its 2006 edition, in the chapter of Biology of the Construction, expressly mentions the placement of beds based on geo-pathogenic influences. Subsequent editions do not include this chapter, inferring the need for new studies on the preventive quality of architecture from a health perspective. These studies should contain, as a primary criterion, the importance of recovery rest for humans.

Usually, a person remains in a place for more than 2 years, with a daily frequency of 8 h in the same place in a horizontal, static, and unconscious way. This accumulated potential risk in bedrooms is the fundamental field of application [64], based on the conviction that any methodical progress, even a small one, will illuminate the situation about the influence of the natural environment on human beings. Therefore, the influence of the geomagnetic field on areas of rest in residential buildings has been studied [65]. Given the existence of different types of dwellings and different modes of use, studies adapted to each type are required [66] and must distinguish the geomagnetic effects from magnetism caused by electrical energy in the building [67]. In turn, as already noted, people react very differently to external influences and in their sensitization to a place, although it may take as long as 2 years in the same place for these effects to be detected. These effects can also interact with other radiations of artificial origin [68].

The complex relationship between health and the environment extends its complexities to the health sector and extends the urban planning field to residential design. Our contribution as designers is one more link in a chain of schemes in different professional disciplines that seek healthier buildings. Studies in other geographical areas [69] on the influence of the geomagnetic field and the causes of its variability in absolute terms are required. Therefore, we propose an analysis of geomagnetic field variation with respect to the adopted dosimetry and establish the causes related to residential design.

## 3. Methodology

This paper analyses the levels of variability with respect to the baseline values of the terrestrial magnetic field within a building, in an attempt to detect zones with a greater impact. The scope of this article is limited to the study of the geomagnetic field in the zones where the habitants rest, for the purpose to compare the variability with dosimetry of reference and the significant statistic factors. The geomagnetic field is around 0 Hz, although different frequencies between 16 Hz and 2 kHz are recorded in each measurement. An attempt is made to evaluate the natural influence in spaces used by people in the same place with extended stays. We start from the values obtained close to 1000 nT (1 µT) of variability associated with the statistical factors analysed within the same building. The process followed in this investigation is as follows:

- Analysis of context and reference dosimetry on the influence of the geomagnetic field variability in the human being and its causes that may be related to the design of residential buildings.
- Selection of a representative case study to obtain data from the geomagnetic field at different points of a building.
- Selection of the appropriate measurement equipment for the definition of the measurement process and data collection.
- Analysis of results in the time domain.
- Analyses of results in frequency mode with the Fourier transform.
- Comparison of trends between the time and frequency modes.
- Discussion of statistical results.
- Discussion of significant architectural causes.
- Presentation of residential design schemes.

After describing the context and dosimetry, the method focuses on monitoring bedrooms of different dwellings at varying heights in a residential building for two and a half years, from March 2015 to September 2017. The location is the Spanish city of Pamplona, which has the following geodetic coordinates: Latitude 42°48′21.5″ N, Longitude 1°38′10.4″ W, Altitude 524.5 m [70]. A residential tower with a ground level plus 16 floors and two basement levels was selected. Finished in 1980, it has 80 dwellings between floors one to 16, one dwelling and commercial areas on the ground floor and garages next to the basement facilities.

### 3.1. Measurement Protocol

The data collection was arduous due to the necessary authorizations for access and data protection. All requirements for this study were satisfactorily achieved for 48 dwellings. After obtaining and analysing the project and site management documentation, we visited the common areas of the building and each dwelling together with each owner. The section and all the floors of the building were drawn up according to distribution, use, structure, and facilities. Each type of use pertaining to the bedrooms was superimposed by layers, and the overlap along the same vertical (Z direction) was analysed for all floors. The duration of the measurement in each dwelling was 24 h. Although other studies have used other geomagnetic field measurement systems in bedrooms [71,72] we selected the NFA1000 3D geo-magnetometer by Gigahertz [73], with a non-electroconductive tripod with a terrestrial magnetic field sensor and a compass to obtain the vertical cross-section.

A measurement protocol was defined to distribute the equipment in the building in all possible situations and heights. Reference was made to Carl Friedrich Gauss’s initial experiment (1777–1855) [50]. The arrangement of uses, both by floor and height, was analysed and plotted to know their dimensional, structural, constructive, and installation characteristics. The equipment was placed inside each dwelling at the same height over the pavement and with the same magnetic orientation in a north-south direction. The use of an additional magnetic field sensor with frequencies from 0 Hz to 100 Hz is required. The duration of the measurement and the distance to the enclosures were one metre to minimize the influence of the electrical installation on the measurement of artificial magnetism to 50 Hz.

The equipment records one measurement every 0.1 s. Therefore, after the interval of 24 consecutive hours, 864,000 data points were obtained from each dwelling. After processing the information with the program NFS A soft 16.1 and collecting the measurements of all the dwellings, the recording interval was set at 17.5 s, yielding 4872 data points for each interval. Subsequently, we had to match each data point to the same time frame in all dwellings. Thus, for the analysis in Time mode, we obtained 233,898 data points for the 48 dwellings, which exceed the required sample size of 6231 data. The analysis was performed using a 95% confidence interval, for a normal standardized distribution and a range amplitude of FE = 0.1 µT and the standard deviation was calculated. After compiling the data, the precise statistical model was applied as a function of the variables chosen, and the analysis was conducted in Time mode.

### 3.2. Frequency Mode

For the analysis in Frequency mode, the same measurement protocol was followed with the equipment, although the processing was different, since the values of each dwelling were grouped into eleven frequencies, given that the variability within each frequency was evaluated, among all the dwellings. After compiling the data, the precise statistical model was applied as a function of the variables for the analysis in Frequency mode.

The study in frequency mode was specified by the Fourier transform, which is a mathematical transformation used to pass signals between the time domain and the frequency domain, and vice versa [74,75]. It is expressed mathematically in the following way:

$$X\left(\omega \right)=\int_{-\infty }^{\infty }x\left(t\right)\cdot e^{-j\cdot \omega \cdot t}\cdot \mathrm{dt}$$

$$x\left(t\right)=\frac{1}{2\pi }\cdot \int_{-\infty }^{\infty }X\left(\omega \right)\cdot e^{j\cdot \omega \cdot t}\cdot d\omega$$

where $j=\sqrt{-1}$, t is the time in seconds, x(t) is the signal in the time domain, ω is the frequency in radians/second and X(ω) is the signal spectrum x(t) in the frequency domain.

As in our case, the measurement process provides a discrete time signal, which means that we know only the value of the signal for homogeneously separated time instances, which is why it is known as the sampling time ($T_{s}$). For a finite duration, we will use the discrete Fourier transform (DFT), which is defined as follows [76]:

$$X\left[k\right]=\{\begin{array}{cc} \sum_{n=0}^{N-1}x\left[n\right]\cdot e^{-j\cdot \frac{2\pi \cdot k\cdot n}{N}} & 0\leq k\leq N-1\\ 0 & \mathrm{other} \end{array}$$

$$x\left[n\right]=\{\begin{array}{cc} \frac{1}{N}\cdot \sum_{k=0}^{N-1}X\left[k\right]\cdot e^{j\cdot \frac{2\pi \cdot k\cdot n}{N}} & 0\leq n\leq N-1\\ 0 & \mathrm{other} \end{array}$$

where $x\left[n\right]=x\left(n\cdot T_{s}\right)$, n is the index of the samples in the time domain, k is the index of the samples in the frequency domain and N is the total number of samples.

The programmed DFT in Matlab language, version R2015b, was applied, since the recorded signals were finite (duration always less than 24 h) and were obtained using $T_{s}=0.1$ ms.

We will use the DFT to represent the temporal signals in the frequency domain. This reordering of the information can lead to new conclusions when analysing the data reorganized by frequencies. In other words, different trends can be obtained in the comparison of the data if different frequencies are analysed, instead of considering only the time domain.

By transforming the signals to the frequency domain, we can select parts of special importance, eliminate the rest (using advanced filtering techniques) and return to the temporal domain where we will have cleaner signals.

### 3.3. Summary

In summary, this methodology allows us to analyse geomagnetic field variation that may occur:

- Due to the effect of architectural design, by superimposing different uses and spaces between the different floors of the building.
- Due to the arrangement of ferromagnetic elements in the resistant structure, both in foundations and in the slabs of the building.
- Due to the geological disturbance of water streams, wells or faults, in such a way that in a space, one zone will be neutral, without significant alteration of the base value, and another one will present percentages of remarkable increase.

Once the comparative results were obtained, schemes for preventive architectural solutions could proceed. To establish a monitoring methodology, the characteristics were analysed so that the approximation to the results was empirical, taking into account the following aspects:

- Determine the places of prolonged stay, in particular the bedrooms, to compare the variation of measurements between the different dwellings.
- Carry out the measurement in all possible dwellings of the building, locating the measurement points of the 16 residential floors.
- Determine the duration (minimum 24 h) and the location of the measurement in each bedroom according to the overlapping uses of the entire building.
- Increase nocturnal measurements by increasing increments starting between 2 and 4 o’clock in the morning.
- Determine in one metre both the height and the distance of the measuring device to avoid influences of the magnetism contributed by the electrical installation, either on the upper or lower floor, by adjoining dwellings or by electrical wiring embedded in the enclosures.
- Determine different seasons of the year to compare rainy and dry seasons.

In summary, a statistical model was designed that considers all the potential influencing variables at the same time. We looked for a representative sample, in terms of both urbanism and building typology with respect to the facility, structure and construction systems to serve as a comparative model. 

The initial Hypothesis 0 is that geomagnetic field variability, if any, is minimal and close to the daily variability of 100 nT, known and measured in the Ebro Observatory. After applying the descriptive methods to the actual measurements obtained, it can be statistically argued that there is geomagnetic field variability.

Once the statistical significance of each variable was obtained, we compared them with the minimum reference dosimetry threshold close to 1000 nT, to propose design alternatives related to the architectural characteristics of each variable.

## 4. Case Study

This paper is limited to defining recommended parameters for residential uses of permanent dwellings. We focus on the residential typology in general and the high-rise building in particular, since the overlap of different uses is inevitable in this building typology. Other occasional uses are not subject to study, although these methods can be extended to other uses of extended stay such as nursing homes, day care institutions, or hospitals, which can be especially important considering the lower defences of the elderly, children, and patients. The intent is not to establish a classification of buildings but to consolidate a few baseline references for health where new or existing permanent housing is our first objective. The choice of this standard residential high-rise building gives this work a generic and extrapolatable component, useful for further studies. The selected building is a tower with a ground floor and 16 floors that adapts to the basic requirement of typology, availability, and grouping of measurements. It is located on Esquiroz Street, 20 b of the Iturrama neighbourhood of Pamplona.

**Figure 1:**
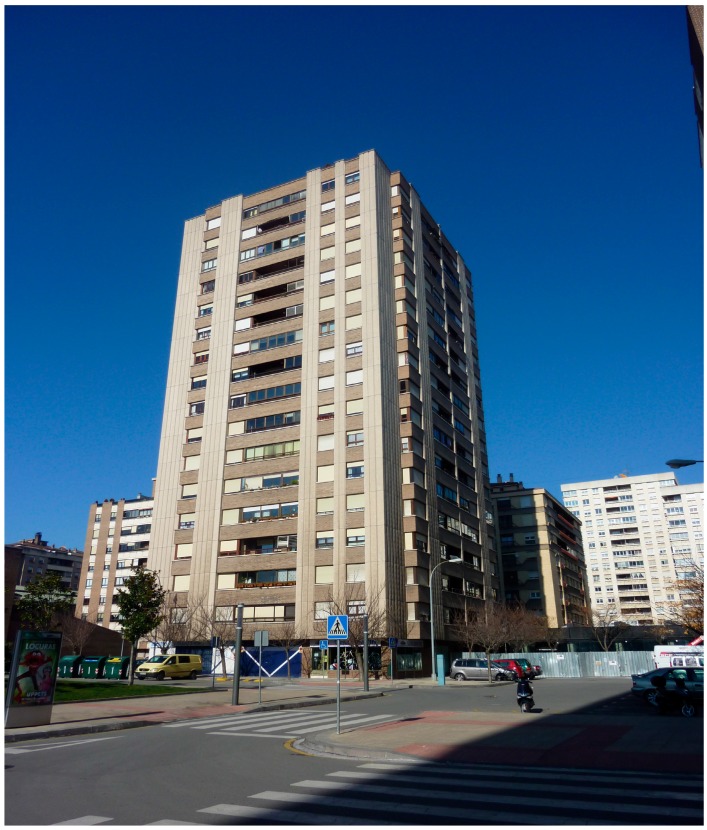
Case study tower’s photo.

Our collective housing is typical on a general level, as an inheritance of the postulates of the Modern Movement. The lack of developed land in the 1960s and the limited time to cover the new residential needs implied that the edification in height, after five floors would be most usual. Pamplona has 90.175 dwellings, which 48.050 within 1940–1980 periods that is approximately 53%. Moreover, the single-family implied 3% vs. 97% collective home [77]. In Spain, we had at our disposal 25,200,000 million homes according to the last census of 2011 [78]. In which 70% are of first home and a 30% as second home. It is rated that 15% of homes are vacant. Moreover the dwellings built between 1940 and 1980, had the highest potential for renovation as they made up 56% of the total. The European research project TABULA, Typology Approach for Building Stock Energy Assessment; establishes different residential typologies for each country [79]. Mainly we can discern between the single-family house and the collective building. The percentage of collective dwellings in Spain gets at 75%, and it is similar to several countries like Italy, Germany, France, Poland, and Sweden, where the portion of this typology is more than 50%. Therefore, our study case is within this group, so it is representative. Figure 1 below shows a tower’s photo.

This urban neighbourhood was planned with large blocks, where interior landscaped spaces alternate with residential buildings with a typological tower shape. It is delimited by the neighbourhood of La Milagrosa to the east and the campus of the University of Navarra to the south. The urban planning and topography facilitate the transition of underground streams from the park of the citadel to this neighbourhood. This building has also been chosen due to the construction date and as representative of structural and construction systems in the last two decades of the twentieth century in Spain. The Ministry of Development of Spain has published a national map with the different levels of the geomagnetic field. In Navarra, values of 45.5 µT are indicated. The Observatory closest to our case study is the Ebro Observatory (Latitude: 40.8417 Longitude: 0.45), where graphs of continuous measurement of the Earth’s static magnetic field can be obtained [80]. In Pamplona, the established value is 45.8 µT, although over time, there are increases, which are measured every ten years. Thus, in 1995, the value was 45.2 µT, in 2005, the value was 45.5 µT, and in 2015, the established value was 45.8 µT.

### 4.1. Geotechnical Case

To analyse the type of land on which the case study building is based, a reference has been made to the geotechnical study of the annexed land located less than 30 m away, performed in December 2002 by the Testing Laboratories of Navarra (n° 8743/02). The overlap between the civic centre and our building with respect to the two projected basements and their proximity supports the similarity of the terrain characteristics, in addition to the geological stability of this neighbourhood and Pamplona in general, with the presence of weathered marl (tufa) at 2.5 m depth. This study included two mechanical test pits and four heavy dynamic penetration tests, as well as precise laboratory tests. The maximum depths reached in the survey were 3 and 3.5 m for the two test pits and between 3.08 m and 3.96 m for the four penetrometer measurements. The layers in descending order are as follows:

- Layer 1: Anthropic fillings up to 1 m deep.
- Layer 2: Sandy-clay gravel from −1.00 m to −1.90 m.
- Layer 3: Clayey-sand gravel from −1.90 m to −2.50 m.
- Layer 4: Weathered marl (Tufa) from −2.50 m to −3.50 m.
- Layer 5: Grey marl substrate from −3.58 m to −3.96 m.

Layers 4 and 5 are rocks in a healthy state, corresponding to the Pamplona marl formation from the Biarritziense (Eocene). It is a massive, very weathered soft rock. It is the recommended layer for foundation support, with an estimated compressive strength greater than 4 MPa. The aggressiveness tests do not determine that the ground can be described as aggressive given the sulphate values of 161 (mg/kg) and 235 (mg/kg).

Regarding the presence of water in the ground, during the field work of the geotechnical study, water seeps were observed at 2.50 m depth of the clayey-sand gravel of layer 3, given that underground streams are high in this area of the city [81]. The occasional presence of water in higher sections of the layers is expected in layers constituted by materials of certain permeability, such as fillings and gravels. These are supported by moderately altered but much less permeable layers. Significant variations in underground streams occur, by season or after the occurrence of heavy downpours. Pumping equipment is recommended as well as waterproofing design in the basement floors.

Looking at the geotechnical study, we can see that first, the land in our study is typical and characteristic of Pamplona; therefore, the presence of ferromagnetic materials does not affect our monitoring. Second, it reinforces that the type of building chosen should be standard so that our work can be extrapolated to other buildings. Third, the presence of filtered water of a pluvial origin at 2.50 m depth implies that during the excavation of the basements of our building, it must have appeared and had to have been channelled. Indeed, it was found that in the second basement, there was a water pumping well that filtered from the surrounding land. On our site, the Pamplona fault is also presented as a geological reference [82].

### 4.2. Building’s Characteristics

**Figure 2:**
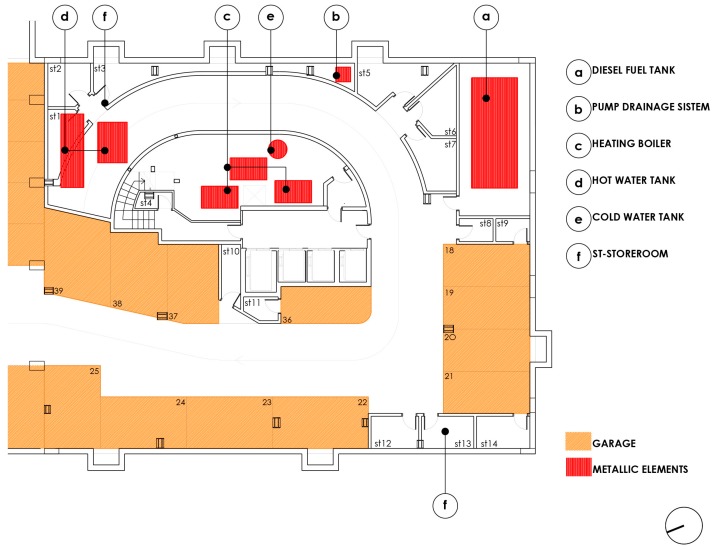
Basement level −2 plan.

The building is a tower with two basement floors, a ground floor and 16 floors (1 to 16), with five dwellings per floor. The dimensions of the floor plan are 29 × 24 m. The central communications core includes three elevators and a forklift, next to the common stairs of the building. The ground floor is intended for pedestrian and road access, three possible commercial areas and housing for the custodian. The parking and the facilities room are located in the basement floors (−1, −2). Figure 2 below shows the floor basement −2, there are, in addition to the parking spaces for vehicles, the boiler room with hot water tanks, and a metal fuel tank. There are three water tanks and three boilers around the central core.

**Figure 3:**
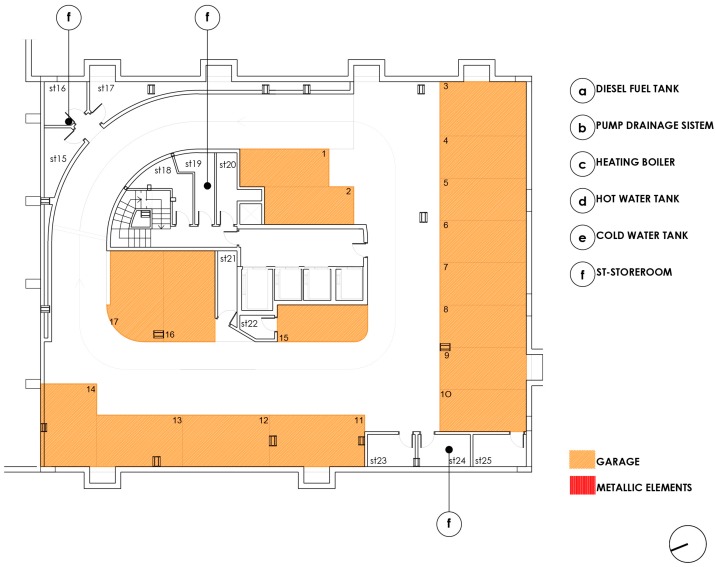
Basement level −1 plan.

Figure 3 above shows the floor basement −1, there are parking spaces and storage rooms on two sides of the perimeter, with the ramp and corridor surrounding the communications core.

**Figure 4:**
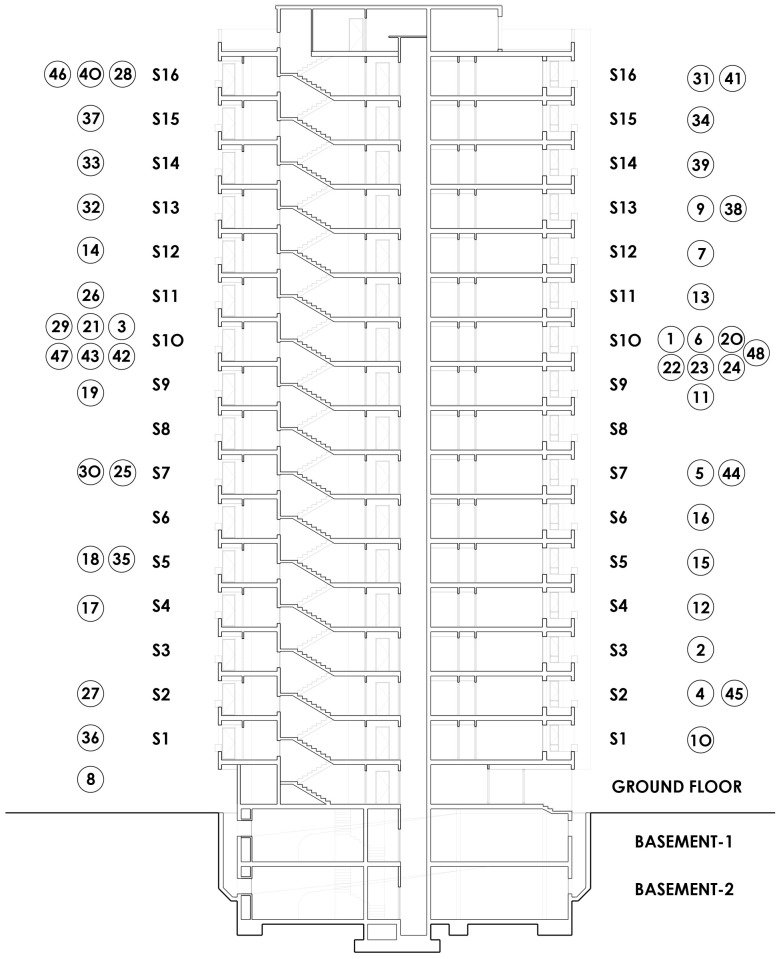
Building’s section.

On the ground floor, the active commercial areas are a bicycle shop-workshop and a vehicle appraisal workshop; the other is a private meeting room. In addition to the custodian’s dwelling, the tower includes 16 elevated floors with five dwelling types (A, B, C, D and E), and the dwellings of floor number 16 are extended to the rooftop in the form of an attic. The dwellings of all floors are identical, except for some size modification, which occurs in some dwellings by exchanging a bedroom. Dwellings A and E have the same layout, as do B and D. Type C is different. Access to the dwellings is located in the core area next to the elevators and the stairway. The north-south orientation in the direction of the longer side of the rectangle of the plot means that dwelling A has East and South facades, dwelling B faces to the South and West, C to the west, D to the West and North and E to the North and East. Figure 4 below shows the building’s section with the measure points for each floor.

From the documentation of the project and the end of completion, the considerations closest to the object of our study, such as the foundations, the structure and the construction elements, are extracted. The structure of the building rests on a foundation based on footings centred with bracing beams supported 7 m deep on the grey marl due to its high strength. The constructive details show a considerable assembly of both the footings and the braces. This solution allows the area occupied by the building to have an area of 50.55% free of reinforcement, given that the foundation occupies a surface of 270 m^2^ with respect to the total surface area of 546 m^2^. The inner columns are made of steel of different dimensions and fire-proofed with sprayed concrete and are the full height of the building. The perimeter columns are made of reinforced concrete up to the reticular slabs of the ground floor. From that level, the columns are metallic until the last floor. The central communication core acts as a structural stiffener and consists of several 28 cm thick reinforced concrete walls. All the slabs are reticular with a 25 + 3 cm edge and an 80 × 80 cm grid, with reinforcing abacus around the columns in the form of solid slab with upper and lower reinforcement. These reinforcing abacuses have been drawn and superimposed on the layouts of the dwellings. The facilities, such as supply, sanitation, electricity, fire, heating, domestic hot water, natural and forced ventilation in basement floors, are the minimum required in a residential building. This description indicates the construction criteria that were widespread in residential buildings at that time, far from the higher standards currently required. However, concerns such as those raised in this study have not yet evolved or been taken into account in the architectural design.

The floor plans and a section of the building are included, indicating the number of each measurement made in a dwelling. This process was followed for each of the 48 dwellings. 

## 5. Results

**Figure 5:**
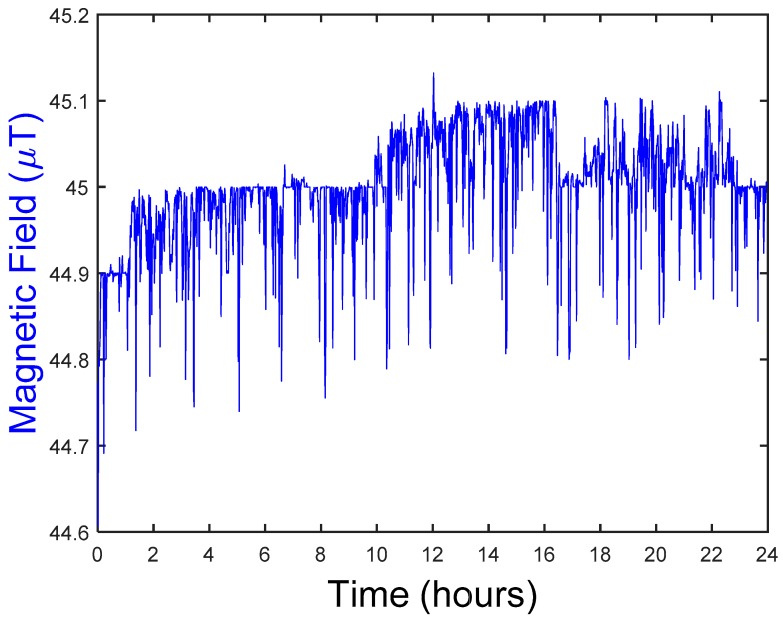
Example measurements in dwelling n° 47. Time.

**Figure 6:**
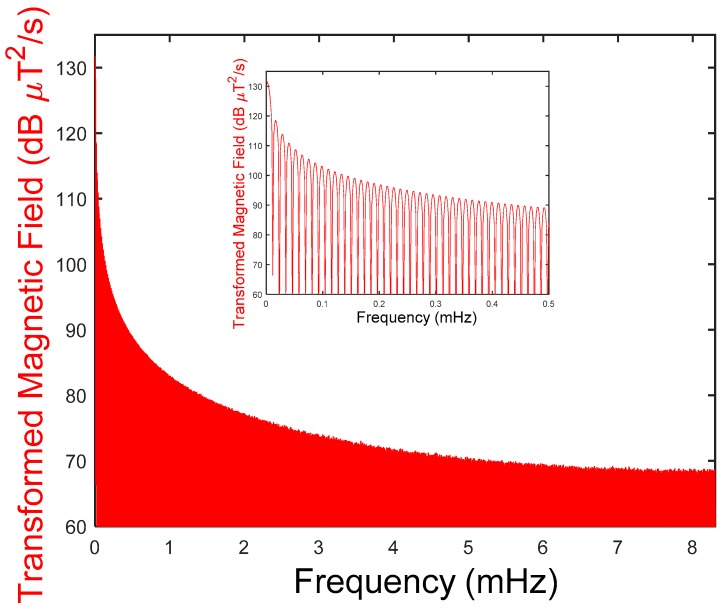
Measurements dwelling example n° 47. Frequency.

In this section, the results from the measurements explained below are shown. First, a complete record obtained directly by the measurement equipment is shown, that is, the raw data as a function of time. Specifically, Figure 5 below shows the magnetic field at location number 47 taken by geo-magnetometer 3D-NFA-1000 for 24 h, which provides a total of 846,000 values. This information can be transformed to the frequency domain using the Fourier Transform, as explained above. This provides a reordering of temporal data that can be very useful for identifying patterns and correlations. Specifically, Figure 6 shows the magnitude of the Fourier transform of the magnetic field data taken by geo-magnetometer 3D-NFA-1000 at location number 47 over 24 h.

This information in the time and frequency domains, together with the rest of the measurements taken in each of the locations (and their transformations to the frequency domain), will be analysed statistically to obtain adequate comparison parameters to formulate the conclusions of this study.

The figure presents the temporal evolution of the magnetic field.

The figure presents the magnitude of the Fourier transformation to the frequency domain of the temporal evolution of the magnetic field in logarithmic scale (decibels, dB). Inset: A detailed display of the lower frequency part of the Fourier transformation where the values are more relevant.

### 5.1. Statistical Variables in Time Mode

Based on its qualitative or quantitative categorization, we have the following types of variables:

- Continuous Quantitative Variables (QTC):
  P0: The measurements of each dwelling are themselves a variable, which allows a descriptive method to be applied, and as a dependent variable, it can be compared with the independent variables. Within each dwelling, we have measurements every 0.1 seconds for 24 h.
  P9 FLOW: This variable is established based on the annual rainfall in the plot occupied by the building, according to the monthly and annual rainfall in Pamplona. A coefficient is assigned in each month, proportional to the mean rainfall.
- Qualitative Variables (QL):

These are defined based on the position of the equipment tripod in relation to the uses of the building, whether a garage or facility rooms located in the basement floors, applying the same criteria for structural elements of floors and foundations. For the correct definition of these variables and after obtaining the project plans and completion plans, new plans have been made for levels that are different in terms of distribution, structure and facilities. After the overlapping of all the layers, the variables corresponding to each monitoring point could be established.

- Nominal Qualitative and Dichotomous Variables (QLND). We create a binary response (0: yes/1: no) as follows:
  P1 ABACUS: Overlapping with structural abacus around the metallic columns of the reticular slab.
  P2 GARAGE: Overlapping of the garage space with any of the two basement floors.
  P3 MMET: Overlapping of metallic elements: diesel fuel tank, cold or hot water tank, heating boilers, and underground water pumping equipment.
  P7 DAY-NIGHT: 8–21 h as 0: Yes Night: 21–8 h as 1: No. In the database, all the measurements of the dwellings have been compared in the same time frame, assigning 12 h for the day and 12 h for the night.
  P10 FOUNDATION: Overlapping between the foundations of the building, either footings or braces, with the position of the NFA equipment. The code is 0: Yes, 1: No
- Nominal Qualitative Polychotomous Variables (QLNP). These give several categories by variable and are as follows:
  P4 HEIGHT: Floor number in height: 0 to 16. From ground floor = 0 to floor 16 = 16.
  P5 SITUAC: Dwelling location at the floor of the building: five per floor: A: 0; B: 1; C: 2; D: 3; and E: 4.
  P6 MONTH: Month during the measurement: 0 to 11. January: 0; February 1; March: 2; April: 3; May: 4; June: 5; July: 6; August: 7; September: 8; October: 9; November: 10; and December: 11.
  P8 TIME OF DAY: From 0 to 23 h. The comparison was made in the database every 17.56 seconds for all the dwellings so that they coincide.

### 5.2. Statistical Variables in Frequency Mode

Regarding the variables in Frequency mode that must intervene based on their qualitative or quantitative categorization, all the qualitative ones related to the temporal mode are excluded from those considered in Time mode. They are P6, P7, and P8; the rest of qualitative variables are maintained with the same characteristics. Considering the basic rule of data comparison in the same frequency band, the quantitative variable P0 acts as a dependent, has been divided into eleven values, which correspond to the analysis of eleven frequencies in each dwelling according to the Fourier analysis. Therefore, we have eleven statistical evaluations corresponding to each of the 48 dwellings for the same frequency. Through the program Stata, version 12, according to the established hypotheses, with the described programming, the method is applied in Frequency mode. This is the same for each of the eleven statistical comparisons, which group the values of each dwelling in the same frequency. All the statistical numerical results, in both time mode and frequency mode, obtained and collected in Table 1, Table 2 and Table 3, allow a direct reading to extract the most significant components that are related to the dependent quantitative variable, with respect to the following independent qualitative variables:

P1 ABACUS: Overlapping with structural abacus around the metallic columns of the reticular slab on each floor of the building.
P2 GARAGE: Overlapping of garage space in any of the two basement floors.
P3 MMET: Overlapping of metallic elements described and located in the two basement floors.
P9 FLOW: Based on the annual rainfall in the plot occupied by the building, the statistical correlation is established, and the mean monthly values of all the months of the year are incorporated. Although the correlation coefficient is weak (−0.1891), less than 0.3, we cannot ignore that this variability of 1720 nT is greater than the reference value of 1000 nT, between the driest month (44,220 nT) and the wettest month (42,500 nT).
P10 FOUNDATION: Overlapping with building foundation elements, either reinforced concrete footings or braces.

To compare the means and derive inferences, the statistical properties of the data for all dwellings needed to be standardized. According to the results of the normality tests of the quantitative variables, we assume that they are normal and we establish the appropriate analysis methods for dependent and independent variables for both Time and Frequency modes.

- ANOVA: Analysis of variance.
- Means: Comparison of Means.
- Corr: Correlation.

Below are the statistical tables of the variables

- Table 1: Statistical method in Time mode.
- Table 2: Statistical method in Frequency mode.

## 6. Discussion

After the application of the described methods and after obtaining the results in the Time and Frequency modes, we proceed to indicate the main criteria based on the statistical significance and the limit value defined in the 1000 nT dosimetry, which is adopted as the reference value.

First, after checking the normality of the P0 variable, we observe the relationship between the P0 measurements and the presence of structural abacus P1, which describes the higher density of steel reinforcements in the slabs of the building, due to the greater concentration of reinforcement around metal columns that a reticular slab requires.

Table 3 below shows the relationship between P0 and P1-structural abacus, which indicates a variability of 775 nT between the binary criteria 0: Overlapping of structural abacus and 1: No overlap. The value obtained in 0 is 42,747 nT, and the value in 1 is 43,522 nT, with a *p*-value of 0.005.

**Figure 7:**
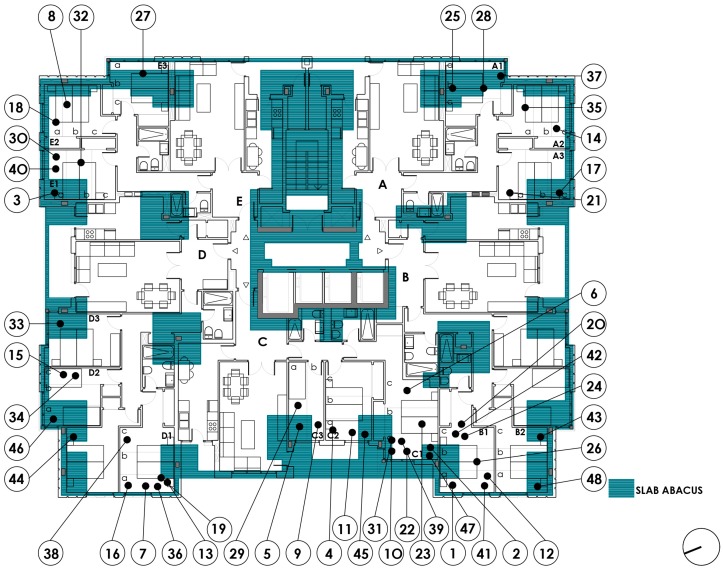
Floor plan dwellings type 1 to 16. Overlapping reticular slab abacus.

Figure 7 below shows the measurements of the bedrooms in each dwelling along the vertical cross-section of a structural abacus. All the slabs have identical structural characteristics, as has been inferred from the project and construction plans. All the measurements obtained over the abacus are graphically arranged above the value 0, whereas the measurements that are not over an abacus and therefore have a lower concentration of reinforcement are arranged over the value 1. Although there is statistical significance, the difference of 775 nT is less than the reference value. We note that there is a reduction of the geomagnetic field due to a higher concentration of reinforcement.

Second, Table 4 below shows the relationship between all the P0 measurements and those with a parking space (P2) in the two basement floors of the building. To determine the part of the bedroom that was in the vertical plane of a parking space, we analysed the uses by redrawing all the different floors, graphically superimposing all the information and visiting each dwelling. 

The relationship between P0 and the variable P2-Parking Space shows a variability of 1548 nT between the binary criteria 0: Overlapping with parking space and 1: No overlap. The value obtained in 0 is 44,315 nT, and the value in 1 is 42,767 nT, with a *p*-value of 0.004. The difference of 1548 nT is greater than the reference value.

**Figure 8:**
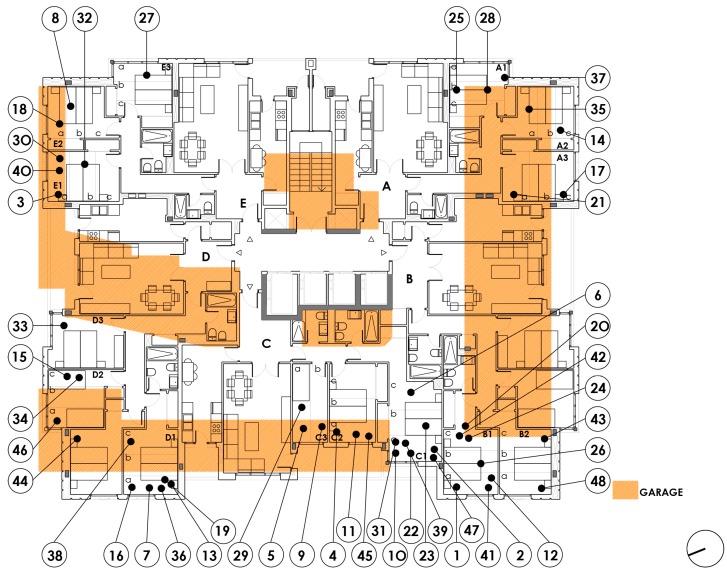
Floor plan dwellings type 1 to 16. Overlapping garages.

Figure 8 below, shows the measurements of the bedrooms for each dwelling with a parking space, either in basement −2 or basement −1. All the measurements obtained on the parking space are graphically arranged over the value 0, whereas the measurements that are not on a parking space are arranged over the value 1. It follows that where there is a parking space, the value of the geomagnetic field is higher.

Third, Table 5 below shows the relationship between all the P0 measurements and the P3 variable. The relationship between the P0 measurements and the variable P3-overlapping with metallic masses points to a variability of 932 nT between the binary criteria of 0 and 1, being 0: Overlapping with metallic mass and 1: No overlap, with a *p*-value of 0.004. Nevertheless, the difference of 932 nT is similar to the reference value. We attribute the increase of the geomagnetic field to the existence of a metallic mass. The value obtained at 0 is 44,000 nT and the value at 1 is 43,068 nT.

**Figure 9:**
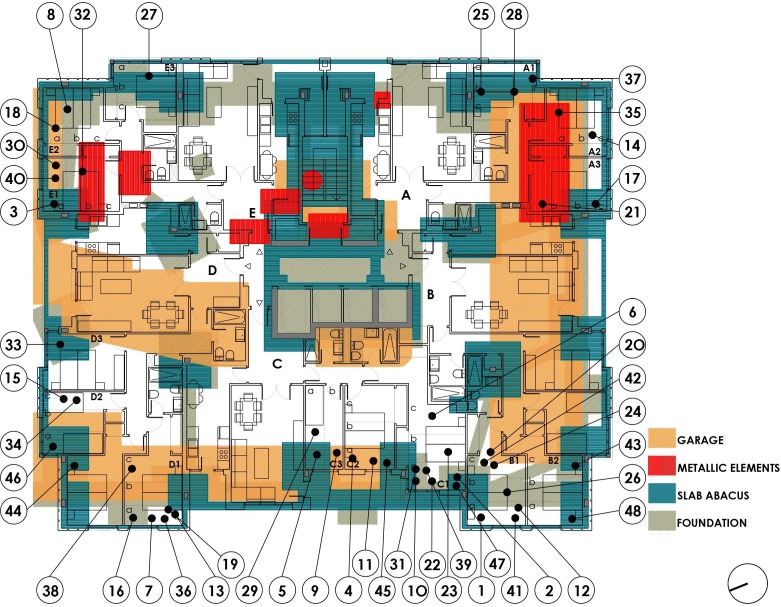
Floor plan dwellings type 1 to 16. Overlapping all metallic elements.

Figure 9 below shows the measurements of the bedrooms for each dwelling with a metallic mass, either in the basement −2 or in the basement −1.

Fourth, in Table 6, we see the relationship between P0 and P7, as a variable that distinguishes between 0: day and 1: night. There is no statistical significance, with *p* = 0.358 and a value of 35 nT produced, very far from the reference value of 1000 nT Therefore, there is no variability between day and night.

Fifth, Table 7 shows the relationship between all the P0 measurements and the variable P10-Overlapping with foundation, which indicates a variability of 499 nT between the binary criteria 0: Overlapping with foundation and 1: No overlap. The value obtained in 0 is 43,010 nT and the value in 1 is 43,509 nT, with a p-value of 0.003. However, the difference of 499 nT is less than the reference value. There is also a reduction of the geomagnetic field due to a higher concentration of reinforcement.

**Figure 10:**
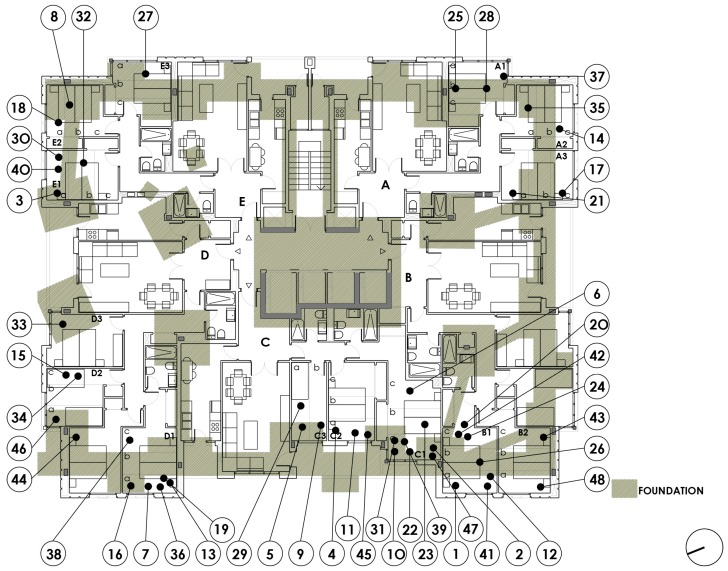
Floor plan dwellings type 1 to 16. Overlapping foundation.

Figure 10 below shows the foundation’s elements, either reinforced concrete footings or braces.

Sixth, Table 8 below shows the relationship between all measurements P0 and variable P9, which estimates the monthly presence of underground streams under the building from the city’s monthly rainfall flow, since a monthly coefficient has been established. The relationship between the P0 measurements and the variable P9-Monthly Rainfall indicates a variability of 1720 nT between the monthly extreme coefficients of the rainfall index. The value obtained for the driest month, with a coefficient of 0.59 for July, is 44,220 nT and the value for the wettest month, with coefficient of 1.34 for November, is 42,500 nT.

In the linear regression, we have statistical significance between P0 and P9, with a correlation coefficient of −0.189, which can be classified as between medium and weak, given that the mean value is 0.3. The negative value indicates that the value decreases with increasing rainfall, which confirms the SBM criterion regarding a lower geomagnetic field value with a greater presence of water [83].

**Figure 11:**
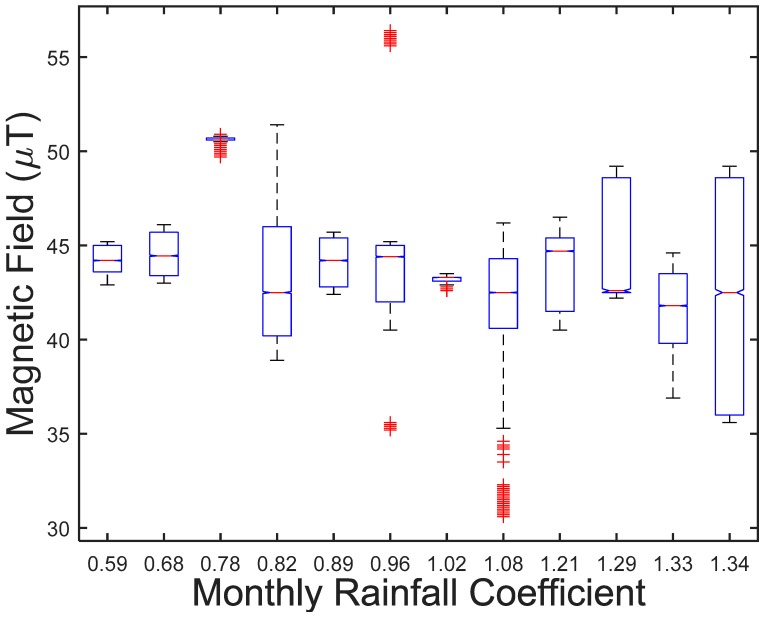
Monthly rainfall coefficient.

Figure 11 shows the relationship between the measurements grouped by monthly coefficients ranging from 0.59 to 1.34. The monthly coefficients are as follows: January: 1.02; February: 0.89; March: 0.96; April: 1.33; May: 1.08; June: 0.82; July: 0.59; August: 0.68; September: 0.78; October: 1.21; November: 1.34; and December: 1.29. 

Variability is best observed in Time mode, although the same trends are confirmed in Frequency mode. They point to a statistical significance because the values are higher than the established reference values of 1000 nT.

Regarding the differences with the variables analysed, P1-structural abacus, P4-height, P5-dwelling location, P6-month of execution, P7-day-night, P8-time of day and P10-Foundations are not statistically significant, or if they are, their values are lower than the reference dosimetry. Table 9 below summarizes the variables P2, P3, and P9 with statistical significance and geomagnetic field variability close to or greater than 1000 nT.

## 7. Conclusions

Our study was able to establish significant statistical associations in relation to the reference dosimetry value of 1000 nT, between the dependent variable P0 and three independent variables, P2-Garage, P3-Metallic masses, and P9-Monthly Rainfall, as previously expected.

The geomagnetic field variability of the rest of the variables does not reach the minimum required dosimetry, although variables such as P1-structural abacus in the slab (775 nT) and P10-foundations (499 nT) obtain statistical significance.

We can conclude that the causes of architectural components that can influence, to a greater or lesser extent, geomagnetic field variability are as follows:

- The arrangement of parking spaces in the basement floors of the building.
- The arrangement of metal masses in the basement floors of the building.
- Variability in storm water due to the flow of underground streams.

Consequently, with this study, it is advisable for the people who occupy the buildings to adopt preventive health measures. For this reason, a series of urban planning and design recommendations for the residential building in the professional architectural field are suggested. These recommendations from our field can provide clues to a global solution and in turn allow savings in public health systems [84].

From the urban point of view, several pathways related to this challenge can be established according to the degree of intervention and the type of planning to be developed. Therefore, general use recommendations at the General Plan level and particular recommendations at the development level for a specific area are suited for this study. In order of importance, when defining criteria for the distribution of detailed uses, it is advisable to request information on existing underground streams, to locate green areas, car parking and roads and to assign the driest lands to residential developments.

On the second level, if there is an urban design with a partial plan, while the constraints may be greater, we always have the possibility of locating residential lands and/or buildable projects in the areas with fewer underground streams. Likewise, it is necessary to take into account the location of underground parking spaces in construction-free areas, for example, in courtyards.

At the third level, when we define a detailed study where the possibilities of changing uses are reduced, we still have a margin of application with the building’s patios and the location of underground parking. From the point of view of architectural design, it is not difficult to include the first two recommendations relating to underground car parking and the overlapping of metal masses in lower floors.

Finally, in regard to designing a building, we know how to synthesize the most relevant issues that as architects we want to express. After the necessary collection of programming, urban and normative data, and no less necessary communication with the client, we must analyse how to define an idea and establish a project process that can navigate this complex creative world, until generating a new reality. Our recommendations can be easily inserted in the design process.

Therefore, the first recommendation is that the criterion of the geomagnetic analysis should not interfere with the initial idea because it should not formally condition us, but rather, it should be incorporated when we define the structural skeleton of the building and when we distinguish the resulting uses. It is time to incorporate these analyses so that bedrooms do not overlap with garages and metal masses, especially at the surface occupied by beds.

At the same time, it should be noted that delaying the incorporation of these recommendations in the process, when the basic project is defined, for example, can be unpleasant, annoying or unnecessary. Therefore, we recommend not only the adoption of these measures but also their introduction in the right moment, as can be outlined in the preliminary project.

The third recommendation, referring to the knowledge of the underground streams, is more complex, given the difficulty of tracing it, at depths greater even than the geotechnical explorations. It would be advisable to commission a geotechnical study of the site after carrying out the preliminary project to indicate the exploration points where the bedrooms are located.

Analysing the phreatic level and the test pits using sensors and inspecting the site will give us clues to this issue and serve to remind us of the importance of location in architecture. How many times do we plan a building without seeing the location fully? In summary, we must be attentive to this third issue and try to obtain information on the possible route of underground streams for the site. More information can be obtained from evidence in nearby lots or in buildings built with pumping wells.

In addition, with regard to the rehabilitation of existing buildings, we also have the opportunity to apply these recommendations, although to a lesser extent than when we have a new project. We can always relocate the bedrooms or the position of the beds in the same bedroom, depending on the parking spaces and the metallic elements. Regarding the location of underground streams, we can gather information in the same building and check the situation of the water pumping wells.

In conclusion, citizens need new tested paradigms that are associated with the current environmental trends of urban regeneration, and modern urban masters plan to allow the consideration of complete design guidelines where the health and well-being of the user are priorities.

### Limitations of the Study

The architectonic design recommendations of the present study contribute to the understanding of this complicated question using a simple implementation and invite to be applied directly, given that it adds to the preventive health purposes. However, the study has its limitations in various sides; one of them is the lack of more measuring equipment to be used simultaneously in several buildings at the same time, which would have allowed contrasting results between them. 

Another limitation is the necessity to conduct more similar studies concerning buildings and residential field, not just homes, in different building sites and with different characteristics, given it would permit the extension of the existing ample spectrum, such as convalescent homes, daycares, colleges, and student residences.

Another limitation has not knowing the position of the groundwater and terrain flaws within the building, which would have allowed introduction of another dichotomous variable to clarify the association with the rain flow. 

Because of data’s protection, confidential information has not been solicited from the residents in the building, relative to the way of life and state of health.

These limitations will not alter the conclusions of the study and should have required a level of monetary investment which would have resulted insuperable.

At the same time, the architectonic language and prevention’s are usual worldwide, and our indications don’t turn out a problem for its adaptation in the regulatory systems of each country and means may be freed for the sustainability of the sanitary system.

We also identify many opportunities for future investigations in this purview due to the considerable benefits obtained concerning the minimum economic costs the application has.

## Figures and Tables

**Table 1:** Statistical method time mode.

P0	P1	P2	P3	P4	P5	P6	P7	P8	P9	P10
	QLND	QLND	QLND	QLNP	QLNP	QLNP	QLND	QLNP	QTC	QLND
QTC	Means	Means	Means				Means			Means
QTC				ANOVA	ANOVA	ANOVA		ANOVA		
QTC									Corr	

P0: Dependent Variable; P1 to P10: Independent Variables.

**Table 2:** Statistical method frequency mode.

P0	P1	P2	P3	P4	P5				P9	P10
	QLND	QLND	QLND	QLNP	QLNP				QTC	QLNP
QTC	Means	Means	Means							Means
QTC				ANOVA	ANOVA					
QTC									Corr	

P0: Dependent Variable; P1 to P5 & P9 and P10: Independent Variables.

**Table 3:** Results: P0 by P1: Variability 775 nT between 0 and 1 with *p*-value: 0.005.

P0–P1	Two-Sample *t* Test with Equal Variances-P0, by (P1)—Reticular Slab Abacus					
Group	Obs	Mean	Std. Err	Std. Dev	95% Conf. Interval	
0	73,113	42.747	0.013	3.548	42.721	42.772
1	160,783	43.522	0.010	4.202	43.501	43.542
Combined	233,898	43.279	0.008	4.025	43.263	43.296
Difference		−0.775	0.017		−0.810	−0.740

**Table 4:** Results: P0 by P2: Variability 1548 nT between 0 and 1 with *p*-value: 0.004.

P0–P2	Two-Sample *t* Test with Equal Variances-P0, by (P2)—Garages					
Group	Obs	Mean	Std. Err	Std. Dev	95% Conf. Interval	
0	77,352	44.315	0.013	3.768	44.289	44.342
1	156,546	42.767	0.010	4.050	42.747	42.787
Combined	233,898	43.279	0.008	4.025	43.263	43.296
Difference		1.548	0.017		1.513	1.582

**Table 5:** Results: P0 by P3: Variability 932 nT between 0 and 1 with *p*-value: 0.004.

P0–P3	Two-Sample *t* Test with Equal Variances-P0, by (P3)—Metallic Masses					
Group	Obs	Mean	Std. Err	Std. Dev	95% Conf. Interval	
0	53,061	44.000	0.010	2.490	43.979	44.021
1	180,837	43.068	0.010	4.352	43.048	43.088
Combined	233,898	43.279	0.008	4.025	43.263	43.296
Difference		0.932	0.019		0.893	0.971

**Table 6:** Results: P0 by P7: Variability 35 nT between 0 and 1 with *p*-value: 0.358.

P0–P7	Two-Sample *t* Test with Equal Variances-P0, by (P7)—Day-Night					
Group	Obs	Mean	Std. Err	Std. Dev	95% Conf. Interval	
0	116,021	43.262	0.011	2.490	43.979	44.021
1	117,877	43.297	0.011	4.352	43.048	43.088
Combined	233,898	43.279	0.008	4.025	43.263	43.296
Difference		−0.035	0.016		−0.067	−0.002

**Table 7:** Results: P0 by P10: Variability 499 nT between 0 and 1 with *p*-value: 0.003.

P0–P10	Two-Sample *t* Test with Equal Variances-P0, by (P10)—Foundation					
Group	Obs	Mean	Std. Err	Std. Dev	95% Conf. Interval	
0	107,586	43.010	0.010	3.427	42.989	43.030
1	126,312	43.509	0.012	4.459	43.484	43.534
Combined	233,898	43.279	0.008	4.025	43.263	43.296
Difference		−0.499	0.016		−0.531	−0.466

**Table 8:** Regress P0–P9-Monthly Rainfall.

Source	SS	df	MS		Number of obs	233,898
Model	135,554.172	1	135,554.172		F (1,233,896)	8673.69
Residual	3,655,372	0.892	15.6281976		Prob > F	0.0003
Total	3,790,927	0.0723	16.2076772		R-squared	0.0358
					Adj R-squared	0.0358
					Root MSE	3.9533
P0	Coef.	Std. Err.	t	P > |t|	95% Conf. Interval	
P9	−3.531	0.0379	−93.13	0.0003	−3.605	−3.456
cons	46.993	0.0407	1154.52	0.0003	46.913	47.073

Corr P0 P9 (obs = 233,898): −0.1891.

**Table 9:** Statistical Time Significances.

Variables	P2	P3	P9
Variability	1548 nT	932 nT	1720 nT

## References

[1] Augner C., Hacker G.W., Jekel I. (2010). Geopathic stress zones: Short-Term effects on work performance and well-being?. J. Altern. Complement. Med..

[2] Marmot A.F., Eley J., Stafford M., Stansfeld S.A., Warwick E., Marmot M.G. (2006). Building health: An epidemiological study of “sick building syndrome” in the Whitehall II study. Occup. Environ. Med..

[3] La Cifras del Cáncer en España. https://www.seom.org/es/noticias/106525-las-cifras-del-cancer-en-espana-2018.

[4] Gratton L., Scott A. (2017). The corporate implications of longer lives. MIT Sloan Manag. Rev..

[5] Palmer S.J., Rycroft M.J., Cermack M. (2006). Solar and geomagnetic activity, extremely low frequency magnetic and electric fields and human health at the earth’s surface. Surv. Geophys..

[6] Khayat D., Ediciones Temas de Hoy (2011). La Biblia Contra el Cancer.

[7] SCENIHR-European Union (2008). Limitation of Exposure EMF.

[8] Meacham B.J. (2016). Sustainability and resiliency objectives in performance building regulations. Build. Res. Inf..

[9] Malmqvist T. (2008). Environmental rating methods: Selecting indoor environmental quality (IEQ) aspects and indicators. Build. Res. Inf..

[10] Havas M. (2017). When theory and observation collide: Can non-ionizing radiation cause cancer?. Environ. Pollut..

[11] Health Protection Agency (2008). UK Static Magnetic Fields. RCE-6.

[12] Hardell L., Sage C. (2008). Biological effects from electromagnetic field exposure and public exposure standards. Biomed. Pharmacother..

[13] Kavet R., Hooper H.C. (2009). Residential magnetic fields and measures of neutral-to-earth voltage: Variability within and between residences. Health Phys..

[14] (2015). IBN-SBM-2015-Standard of building biology. Baubiologie Maes-SBM 2015.

[15] Belova N.A., Acosta-Avalos D. (2015). The effect of extremely low frequency alternating magnetic field on the behavior of animals in the presence of the geomagnetic field. J. Biophys..

[16] Gmitrov J., Gmitrova A. (2004). Geomagnetic field effect on cardiovascular regulation. Bioelectromagnetics.

[17] Havas M. (2004). Biological effects of low frequency electromagnetic fields. Electromagnetic Environments and Health in Buildings.

[18] Van Deventer T.E., Saunders R., Repacholi M.H. (2005). WHO health risk assessment process for static fields. Prog. Biophys. Mol. Biol..

[19] Kirschvink J.L., Kobayashi-Kirschvink A., Woodford B.J. (1992). Magnetite biomineralization in the human brain. Proc. Natl. Acad. Sci. USA.

[20] (2010). ICNIRP Guidelines for limiting exposure to time-varying electric and magnetic fields (1 Hz to 100 kHz). Health Phys..

[21] World Health Organization (2007). Criteria 238 Environmental Health Criteria 238 Extremely Low Frequency Fields.

[22] Parliamentary Assembly (2011). PACE-Resolution 1815 (2011).

[23] Panda S. (2016). Circadian physiology of metabolism. Sci. Assoc. Adv. Sci..

[24] Dharmadhikari N.P., Meshram D.C., Kulkarni S.D., Hambarde S.M., Rao A.P., Pimplikar S.S., Kharat A.G., Patil P.T. (2010). Geopathic stress: A study to understand its nature using Light Interference Technique. Curr. Sci..

[25] Repacholi M.H. (2003). WHO’s health risk assessment of ELF fields. Radiat. Prot. Dosimetry.

[26] Kushner R.F., Sorensen K.W. (2013). Lifestyle medicine. Curr. Opin. Endocrinol. Diabetes Obes..

[27] Richman R., Munroe A.J., Siddiqui Y. (2014). A pilot neighborhood study towards establishing a benchmark for reducing electromagnetic field levels within single family residential dwellings. Sci. Total Environ..

[28] Schultheiss-Grassi P.P., Wessiken R., Dobson J. (1999). TEM investigations of biogenic magnetite extracted from the human hippocampus. Biochim. Biophys. Acta—Gen. Subj..

[29] Vanderstraeten J., Gillis P. (2010). Theoretical evaluation of magnetoreception of power-frequency fields. Bioelectromagnetics.

[30] Leszczynski D. (2005). Rapporteur report: Cellular, animal and epidemiological studies of the effects of static magnetic fields relevant to human health. Prog. Biophys. Mol. Biol..

[31] Till U., Timmel C.R., Brocklehurst B., Hore P.J. (1998). The influence of very small magnetic fields on radical recombination reactions in the limit of slow recombination. Chem. Phys. Lett..

[32] Sastre A., Graham C., Cook M.R., Gerkovich M.M., Gailey P. (2002). Human EEG responses to controlled alterations of the Earth’s magnetic field. Clin. Neurophysiol..

[33] Grellier J., Ravazzani P., Cardis E. (2014). Potential health impacts of residential exposures to extremely low frequency magnetic fields in Europe. Environ. Int..

[34] Forssén U.M., Ahlbom A., Feychting M. (2002). Relative contribution of residential and occupational magnetic field exposure over twenty-four hours among people living close to and far from a power line. Bioelectromagnetics.

[35] Zhang J., Ding C., Ren L., Zhou Y., Shang P. (2014). The effects of static magnetic fields on bone. Prog. Biophys. Mol. Biol..

[36] Feychting M. (2005). Health effects of static magnetic fields—A review of the epidemiological evidence. Prog. Biophys. Mol. Biol..

[37] Brocklehurst B. (2002). Magnetic fields and radical reactions: Recent developments and their role in nature. Chem. Soc. Rev..

[38] Hore P.J., Mouritsen H. (2016). The radical-pair mechanism of magnetoreception. Annu. Rev. Biophys..

[39] Zaporozhan V., Ponomarenko A. (2010). Mechanisms of geomagnetic field influence on gene expression using influenza as a model system: Basics of physical epidemiology. Int. J. Environ. Res. Public Health.

[40] Azcarate T., Mendoza B., Levi J. (2016). Influence of geomagnetic activity and atmospheric pressure on human arterial pressure during the solar cycle 24. Adv. Sp. Res..

[41] Lipnicki D.M. (2009). An association between geomagnetic activity and dream bizarreness. Med. Hypotheses.

[42] Burch J.B., Reif J.S., Yost M.G. (2008). Geomagnetic activity and human melatonin metabolite excretion. Neurosci. Lett..

[43] Krylov V.V. (2017). Biological effects related to geomagnetic activity and possible mechanisms. Bioelectromagnetics.

[44] Reiter R.J. (1994). Melatonin suppression by static and extremely low frequency electromagnetic fields: Relationship to the reported increased incidence of cancer. Rev. Environ. Health.

[45] Mitsutake G., Otsuka K., Hayakawa M., Sekiguchi M., Cornélissen G., Halberg F. (2005). Does Schumann resonance affect our blood pressure?. Biomed. Pharmacother..

[46] Besser B.P. (2007). Synopsis of the historical development of Schumann resonances. Radio Sci..

[47] Tesla N. (1905). Art of Transmitting Electrical Energy through the Natural Mediums. U.S. Patent.

[48] Balser M., Wagner C.A. (1960). Observations of Earth–Ionosphere Cavity Resonances. Nat. Publ..

[49] Cherry N. (2002). Schumann Resonances, a plausible biophysical mechanism for the human health effects of solar/geomagnetic activity. Nat. Hazards.

[50] Gubbins D., Herrero-Bervera E. (2007). Encyclopaedia of Geomagnetism and Paleomagnetism.

[51] Blakely R. (1998). Introduction to geomagnetic fields. Eos Trans. AGU.

[52] USA NCEI, UK BGS (2015). The USA/UK World Magnetic Model for 2015-2020.

[53] Mozzoni D. (2007). The Changing Geomagnetic Field from the Ionosphere to the Core-Mantle Boundary.

[54] Eliyahu I., Hareuveny R., Riven M., Kandel S., Kheifets L. (2017). 24-h personal monitoring of exposure to Power Frequency Magnetic Fields in adolescents—Results of a National Survey. Environ. Res..

[55] Kaune W.T., Banks R.S., Linet M.S., Hatch E.E., Kleinerman R.A., Wacholder S., Tarone R.E., Haines C. (2001). Static magnetic field measurements in residences in relation to resonance hypotheses of interactions between power-frequency magnetic fields and humans. Bioelectromagnetics.

[56] World Health Organization (2002). IARC monographs on the evaluation of carcinogenic risks to humans. Int. Agency Res. Cancer..

[57] Brown H.A. (1991). A Palaeomagnetic, Geochronological and Palaeoenvironmental Investigaction of Late and Post Glacial Maar Lake Sediments from NW-Europe.

[58] Kotwicki V. (2009). Water balance of Earth. Hydrol. Sci. J.-J. Des. Sci. Hydrol..

[59] Asimakopoulou F.E., Gonos I.F., Stathopulos I.A. (2012). Methodologies for determination of soil ionization gradient. J. Electrostat..

[60] Auken E., Guérin R., de Marsily G., Sailhac P. (2009). Comptes Rendus—Geoscience.

[61] AEMET (2016). Climatic Parameters of the Pamplona Observatory.

[62] (2017). ILFI Living Building Challenge 3.1.

[63] Neufert E. (2006). Arte de Proyectar en Arquitectura.

[64] Tomitsch J., Dechant E., Frank W. (2010). Survey of electromagnetic field exposure in bedrooms of residences in Lower Austria. Bioelectromagnetics.

[65] Swanson J., Kheifets L. (2012). Could the geomagnetic field be an effect modifier for studies of power-frequency magnetic fields and childhood leukaemia?. J. Radiol. Prot..

[66] Baris D., Linet M.S., Tarone R.E., Kleinerman R.A., Hatch E.E., Kaune W.T., Robison L.L., Lubin J., Wacholder S. (1999). Residential exposure to magnetic fields: An empirical examination of alternative measurement strategies. Occup. Environ. Med..

[67] Kaune W.T., Davis S., Stevens R.G., Mirick D.K., Kheifets L. (2001). Measuring temporal variability in residential magnetic field exposures. Bioelectromagnetics.

[68] Breus T.K., Binhi V.N., Petrukovich A.A. (2016). Magnetic factor in solar-terrestrial relations and its impact on the human body: Physical problems and prospects for research. Physics-Uspekhi.

[69] Watanabe Y., Cornélissen G., Halberg F., Otsuka K., Ohkawa S.I. (2001). Associations by signatures and coherences between the human circulation and helio- and geomagnetic activity. Biomed. Pharmacother..

[70] Navarra G. (2011). Coordenadas Geodésicas ETRS89 Pamplona (PAML).

[71] Vandermeulen D., Vercauteren C., Weyn M. Indoor Localization Using a Magnetic Flux Density Map of a Building Feasibility Study of Geomagnetic Indoor Localization. Proceedings of the Third International Conference on Ambient Computing, Applications, Services and Technologies.

[72] Berkvens R., Vandermeulen D., Vercauteren C., Peremans H. (2014). Feasibility of geomagnetic localization and geomagnetic RatSLAM. Int. J. Adv. Syst. Meas..

[73] (2016). Gigahertz Solutions NFA 1000 and NFA Soft 16.1..

[74] Bracewell R.N. (1965). The Fourier Transform and Its Applications.

[75] Percival D.B., Walden A.T. (1993). Spectral Analysis for Physical Applications: Multitaper and Conventional Univariate Techniques.

[76] Oppenheim A.V., Schafer R.W., Buck J.R. (1999). Discrete Time Signal Processing.

[77] Monge-Barrio A., Sánchez-Ostiz Gutierrez A. (2018). Passive Energy Strategies for Mediterranean Residential Buildings. Facing the Challenges of Climate Change and Vulnerable Populations.

[78] Estadística I.N. (2011). De Censo de Población y Vivienda.

[79] Loga T., Stein B., Diefenbach N. (2016). TABULA building typologies in 20 European countries—Making energy-related features of residential building stocks comparable. Energy Build..

[80] Marsal S., Solé J., Curto J. (2016). Observaciones Geomagnéticas 2016.

[81] Del Valle J. (1974). El Yacimiento Potásico del Perdón.

[82] Vergés J. (2003). Evolution of the oblique ramp systems of the southerm Pyrenees: The Segre and Pamplona faults. Bol. Geol. y Minero. Span..

[83] Maes W., Mierau M., Haumann T., Maes B. (2015). Framework conditions for technical measurements. SBM-2015.

[84] Kandel S., Swanson J., Kheifets L. (2016). Health-Economics analyses applied to elf electric and magnetic fields. Risk Anal..

